# GRK2 differentially regulates FcεRI and MRGPRB2-mediated responses in mast cells

**DOI:** 10.3389/fimmu.2023.1155777

**Published:** 2023-03-29

**Authors:** Monica Thapaliya, Hydar Ali

**Affiliations:** Department of Basic and Translational Sciences, University of Pennsylvania School of Dental Medicine, Philadelphia, PA, United States

**Keywords:** MRGPRX2/MRGPRB2, GRK2, itch, anaphylaxis, mast cells, FcεRI, STAT5

## Abstract

In addition to high-affinity IgE receptor (FcεRI), a subtype of mouse mast cells (MCs) expresses a G protein-coupled receptor known as Mas-related G protein-coupled receptor (GPCR)-B2 (MRGPRB2; human ortholog MRGPRX2). GPCR kinase 2 (GRK2) is a Serine/Threonine kinase that phosphorylates GPCRs to promote their desensitization and internalization. We previously showed that silencing GRK2 expression in mouse bone marrow-derived MCs (BMMCs) blocks IgE-mediated degranulation. Compound 48/80 (C48/80), substance P (SP) and LL-37 cause degranulation in human and mouse MCs *via* MRGPRX2 and MRGPRB2, respectively. We also reported that C48/80 and SP cause desensitization and internalization of MRGPRX2, but LL-37 does not. Here, we generated mice with MC-specific deletion of *Grk2* (*Cpa3Cre^+^/Grk2^fl/fl^*) to determine its role on IgE-mediated responses and to assess whether it differentially regulates degranulation in response to LL-37, C48/80 and SP. Absence of GRK2 substantially inhibited IgE-mediated tyrosine phosphorylation of STAT5, calcium mobilization, and degranulation in mouse primary lung-derived MCs (PLMCs). By contrast, peritoneal MCs (PMCs) from *Cpa3Cre^+^/Grk2^fl/fl^* mice demonstrated significant enhancement of degranulation in response to C48/80 and SP, but not LL-37. Deletion of *Grk2* in MCs attenuated IgE-mediated passive cutaneous anaphylaxis (PCA) and itch but not passive systemic anaphylaxis (PSA). Surprisingly, PSA was significantly reduced in *Mrgprb2^-/-^* mice. These findings suggest that GRK2 contributes to PCA and itch but not PSA. By contrast, GRK2 desensitizes MRGPRX2/B2-mediated responses to C48/80 and SP but not LL-37. However, IgE-mediated PSA likely involves the activation of MRGPRB2 by LL-37 or a similar agonist, whose function is resistant to modulation by GRK2.

## Introduction

Mast cells (MCs) are tissue-resident immune cells that are responsible for allergic disorders such as anaphylaxis, atopic dermatitis and asthma (1, 2). Cross-linking of high-affinity IgE receptor (FcεRI) by allergens on MCs results in the release of prestored inflammatory mediators followed by newly synthesized mediators that are primary drivers of these allergic disorders (2). G protein-coupled receptor (GPCR) kinases (GRKs) are Serine/Threonine (Ser/Thr) protein kinases that are best known for their roles in GPCR desensitization (3, 4). We have recently shown that silencing GRK2 expression in mouse bone marrow-derived MCs (BMMCs) results in significant inhibition of IgE-mediated degranulation (5). By contrast, overexpression of GRK2 in rat basophilic leukemia (RBL-2H3) cells results in enhanced IgE-mediated degranulation and cytokine generation without affecting the expression levels of any of the FcεRI subunits (α,β,γ) or IgE-mediated phosphorylation of Fcγ (5). One objective of the present study was to generate mice with MC-specific deletion of *Grk2* to determine if its absence attenuates IgE-mediated passive cutaneous anaphylaxis (PCA), itch and passive systemic anaphylaxis (PSA) *in vivo.*

In addition to FcεRI, a MC subtype predominantly found in the human skin expresses a novel G protein-coupled receptor (GPCR) called Mas-related GPCR-X2, (MRGPRX2, mouse counterpart MRGPRB2) (6, 7). Serhan et al. (8) recently showed that house dust mice antigen causes the release of the neuropeptide substance P (SP) from sensory neurons. This SP then activates cutaneous MCs *via* MRGPRB2, which contributes to the development of atopic dermatitis-like lesion in mouse skin. However, McNeil et al (7), showed that MRGPRB2 does not contribute to IgE-mediated cutaneous anaphylaxis. By contrast, using a pharmacological approach, Oskeritzian et al. (9), showed that release of Sphingosine-1-phosphate from MCs in response to FcεRI transactivates its cognate GPCR in MCs and contributes to PSA. However, the possibility that MRGPRB2 contributes to IgE-mediated PSA and if GRK2 regulates this response has not been determined.

It is generally accepted that agonist-induced phosphorylation of GPCRs by GRKs and the subsequent recruitment of the adapter protein, β-arrestin promotes their desensitization and internalization (3, 10). However, our lab was the first to demonstrate that although the antimicrobial peptide LL-37 activates human MCs *via* MRGPRX2, the receptor is resistant to LL-37-induced phosphorylation, desensitization, and internalization (11). In addition, shRNA-mediated knockdown of GRK2 had no effect on LL-37-induced MC degranulation (11). Subsequent studies from our lab led to the characterization of MRGPRX2 agonists into two groups, termed G protein-biased and balanced agonists. G protein-biased agonists such as LL-37, icatibant and murepavadin activate G proteins and are resistant to desensitization and internalization (11–13). By contrast, balanced agonists such as C48/80 and SP not only activate G proteins but also promote MRGPRX2 desensitization and internalization (14, 15). Here, we also sought to determine if GRK2 modulates degranulation in response to selected G protein-biased and balanced agonists in mouse peritoneal MCs that endogenously expresses MRGPRB2 *in vitro* and to assess if the receptor contributes to IgE-mediated PSA *in vivo.*

## Materials and methods

### Reagents

DNP-specific mouse IgE (SPE-7) and all cell culture reagents were purchased from Invitrogen (Carlsbad, CA). Recombinant mouse interleukin-3 (IL-3) and mouse stem cell factor (SCF) used in the study were obtained from Peprotech (Rocky Hill, NJ). DNP-BSA, p-nitrophenyl-N-acetyl-β-D-glucosamine (PNAG), Ionomycin, Phorbol 12-myristate 13-acetate (PMA), Evans blue dye and protease inhibitor were obtained from Sigma-Aldrich (St. Louis, MO). KIT antibody conjugated with Phycoerythrin (PE), FcεRI antibody conjugated with Fluorescein isothiocyanate (FITC) and Allophycocyanin (APC) were purchased from eBiosciences (San Diego, CA). Anti-mouse LAMP-1 antibody conjugated with Alexa Fluor 647 and anti-mouse CD31 antibody conjugated with Alexa Fluor 488-conjugated were purchased from BioLegend (San Diego, CA). Halt Phosphatase Inhibitor Cocktail was purchased from Pierce Endogen. Antibodies against HRP-labeled goat anti-rabbit IgG, GRK2, β-actin, P-STAT5 (Tyr 694) and STAT5 were purchased from Cell Signaling Technology (Danvers, MA). SuperSignal^®^ Pico plus Chemiluminescent Substrate was from Thermo Scientific (Rockford, IL). Fura-2 AM and Texas Red-conjugated Avidin was purchased from Molecular Probes (Eugene, OR). LL-37, Substance P (SP) and Compound 48/80 (C48/80) were obtained from AnaSpec (Fremont, CA).

### Mice

Pathogen-free cages with autoclaved hardwood beddings were used to house all mice used in the study. *Cpa3Cre* mice were kindly provided by Dr. Stephen Galli (Stanford University, Stanford, CA) (16) and *Grk2^fl/fl^* mice were kind gift from Dr. Hariharan Subramanian (Michigan State University). Mice with MC-specific deletion of *Grk2* (*Cpa3Cre^+^/Grk2^fl/fl^*) were generated by Cre-Lox breeding scheme by crossing *Cpa3Cre* mice with *Grk2^fl/fl^* mice. Absence of *Grk2* in mice were validated at genomic level based on Cre and Flox specific PCR using *Cpa3Cre^-^/Grk2^fl/fl^* mice as respective controls with the primer sets: *Cpa3Cre* Fwd: ACT GTT CAT CCC CAG GAA CC; *Cpa3Cre* Rev: CAG GTT CTT GCG AAC CTC AT; *Grk2*-Flox Fwd: TGA GGC TCA GGG ATA CCT GTC AT; *Grk2*-Flox Rev: CAG GCA TTC CTG CTG GAC TAG. The wild type (WT, C57BL/6), mice used in this study with were obtained from the Jackson Laboratory (Bar Harbor, ME, USA). CRISPR-Cas9 core facility of the University of Pennsylvania generated *Mrgprb2* deficient mice (*Mrgprb2^-/-^*) in the C57BL/6 background. This study was approved by Institutional Animal Care and Use Committee at The University of Pennsylvania and constituted of 8-12 weeks mice.

### Cells

#### Primary lung-derived MCs (PLMCs)

PLMCs were obtained from *Cpa3Cre^+^/Grk2^fl/fl^* and *Cpa3Cre^-^/Grk2^fl/fl^* fresh lung tissue from mice *via ex-vivo* differentiation as described (17). Briefly, lungs were excised and collected in a 15 mL falcon tube with complete media (Gibco Roswell Park Memorial Institute (RPMI) 1640 media with GlutaMAX and 25mM HEPES supplemented with 10% FBS, 5% Non-Essential Amino Acid (NEAA), penicillin (100 IU/mL) and streptomycin (100 µg/mL). They were then washed with PBS once and chopped into fine pieces with scissors. Chopped lungs were then cultured in complete media with β-mercaptoethanol (45.6 µM) supplemented with murine IL-3 (10 ng/mL), and murine SCF (10 ng/mL). After 7-10 days fat like droplets left behind in the culture were strained. The cells were then cultured continuously removing adherent cells 1-2 times/week for a duration of 4-6 weeks. The purity of the MCs were determined by flow cytometry (BD LSR II flow cytometer, BD Biosciences) using anti-KIT-PE and anti-FcεRI-FITC antibodies.

#### Peritoneal MCs (PMCs)

PMCs from *Cpa3Cre^+^/Grk2^fl/fl^* and *Cpa3Cre^-^/Grk2^fl/fl^* mice were isolated and purified as described (12). Briefly, cells were lavaged from peritoneal cavity using Hank’s Balanced Salt Solution (HBSS) supplemented with 3% fetal bovine serum (FBS) and 1mM HEPES in 10ml volume. The cells were then cultured in complete medium same as PLMCs supplemented with murine IL-3 (10 ng/mL), and murine SCF (30 ng/mL). After 48 hours non-adherent cells were removed. Suspension cells were further cultured in fresh medium for 4-8 weeks supplementing IL-3 and SCF once every week. After 4 weeks of culture, alcian/safranin staining was used to confirm that > 90% of cells were PMCs as described (18). The purity of MCs were determined with same flow cytometric procedure as PLMCs.

#### Mast cell staining

Cultured PMCs (5×10^4^) were stained with alcian/safranin as described (18). Images were captured using Nikon E600 microscope at 10X ND image were analyzed using NIS Elements Software. Dermal sheets of ears from *Cpa3Cre^+^/Grk2^fl/fl^* and *Cpa3Cre^-^/Grk2^fl/fl^* mice were stained using Texas Red-Avidin (MCs)and Alexa Fluor 488-conjugated CD31 antibody (endothelial cells) as described previously (18). Briefly, the dermal sheets were fixed in 3.7% paraformaldehyde overnight, blocked with 0.3% triton and 1% BSA in PBS for 30 min, incubated overnight with Texas Red-Avidin and Alexa Fluor 488-conjugated CD31 antibody, washed for 1 hour and mounted with antifade mounting solution. Nikon A1R laser scanning confocal microscope was used to capture the images with a 40X water objective (NA=1.2). Nikon NIS Elements software was used for image analysis.

#### FcεRI and KIT expression

Cell surface receptor expression in PLMCs and PMCs from *Cpa3Cre^+^/Grk2^fl/fl^* and *Cpa3Cre^-^/Grk2^fl/fl^* were analyzed *via* flow cytometry. Briefly, cells (0.4×10^6^) were washed with buffer PBS containing 2% FBS and 0.02% sodium azide and stained with anti-KIT-PE and anti-FcεRI-APC (PMCs)/anti-FcεRI-FITC (PLMCs) for 30 min on ice. Cells were washed and fixed in 1.5% paraformaldehyde solution. BD LSR II flow cytometer (BD Biosciences) was used to acquire flow cytometry data and analyzed by WinList software.

#### Western blotting

To confirm the absence of GRK2, protein lysate was prepared from PLMCs and PMCs from *Cpa3Cre^+^/Grk2^fl/fl^* and *Cpa3Cre^-^/Grk2^fl/fl^* using RIPA cell lysis buffer with protease inhibitor. Protein then was separated in SDS-PAGE (10%), immunoblotted onto PVDF membranes, blocked, and probed with anti-GRK2 antibody (1:1000). SuperSignal^®^ West Femto Chemiluminescent Substrate was used for membrane development following incubation by HRP-conjugated secondary antibody. The membrane was further subjected to stripping and re-probing with β-actin similarly. IgE-primed PLMCs were stimulated with antigen (DNP-BSA) for different time point and lysed and probed with anti-p-STAT5 antibody. The blot was further stripped and probed with total STAT5 and β-actin antibody.

#### Calcium mobilization

IgE primed PLMCs (0.5 × 10^6^) with DNP-specific mouse IgE antibody (1 µg/mL, 16 h) were first washed in HEPES-buffered saline containing 0.1% BSA. Next, the cells were loaded with 1 μM Fura-2 acetoxymethyl ester for 30 min at 37°C. They were further allowed to undergo de-esterification in the buffer for an additional 15 min incubation at room temperature. Cells were stimulated with 10 ng/ml of DNP-BSA at 100 sec. Calcium mobilization data was collected using a Varioskan™ LUX Multimode Microplate Readers (Thermo Scientific). The dual excitation wavelength used were 340 and 380 nm with an emission wavelength of 510 nm.

#### Degranulation

IgE-primed PLMCs (4 × 10^4^/well) with anti-DNP specific IgE antibody (1 µg/mL, 16 h) were incubated with different concentrations of antigen (DNP-BSA, 0 -100 ng/mL) at 37°C for 30 min in a round bottom 96 well plate. To measure total β-hexosaminidase release, unstimulated cells were subjected to lysis in 50 μL of 0.1% Triton X-100. Aliquots (20 μL) of supernatant or cell lysates were transferred into fresh wells and 20 μL of 1 mM p-nitrophenyl-N-acetyl-β-D-glucosamine was added. The reaction was further incubated for 1 h at 37°C and was stopped by adding 250 μL of a 0.1M Na_2_CO_3_/0.1M NaHCO_3_ buffer. The absorbance was measured at 405 nm. For experiments with PMCs, cells (1 × 10^4^/well) were stimulated with C48/80 (10 µg/mL), SP (50 µM) and LL-37 (10 µM) for 30 min at 37°C. For PMA/ionomycin stimulation, PLMCs were exposed to PMA (50 nM for 5 min) and subsequently stimulated with ionomycin (0 – 2 µg/mL) for additional 30 min and β-hexosaminidase release was determined. Percentage degranulation was calculated by diving sample β-hexosaminidase release with total β-hexosaminidase release. For accessing degranulation *via* cell surface lysosomal-associated membrane protein1 (LAMP-1) expression, IgE-primed cells (0.5×10^6^) were stimulated with DNP-BSA (10 ng/mL) for 10 min, washed and incubated with anti-LAMP-1 Alexa Fluor 647 antibody for 1 hour on ice. Flow cytometric assay was used to determine cell surface expression of LAMP-1 as described (18).

#### Passive cutaneous anaphylaxis

For experiments with IgE-mediated anaphylaxis, mice were intradermally injected with DNP-specific IgE (20 ng, 30 µL) into the right or PBS in the left ear/paw (control). After 24 h, mice were challenged with an intravenous injection of 100 µg DNP-BSA in 200 µL PBS containing 1% Evans blue in PBS. For experiment to assess C48/80-induced vascular permeability, mice were intradermally injected with C48/80 (100 ng, 10 µL, right hind paw) or PBS (10 µL, left hind paw) following an intravenous injection of 100 µL of 1% Evans blue in PBS. After 30 min both experimental tissues were collected and weighed. They were then dissolved in 600 µL formamide and incubated at 55˚C for 24 h. For paw tissues, dry weight was recorded after drying at 55˚C for 24 h before formamide incubation. Next, supernatant was collected and measuring the absorbance at 650 nm dye extravasation was determined.

#### Passive systemic anaphylaxis

PSA mediated *via* IgE was performed as previously described (9). Briefly, mice were passively sensitized with 20 µg of anti-DNP-BSA in 200 µl of PBS intraperitoneally. After 24 hours, mice were challenged with 200 µg of DNP-BSA in 200 µl of PBS intraperitoneally. Every 10 min for 60 min and every 30 min after that until 120 min core body temperature was measured with a rectal thermometer (physitemp) and recorded. After 120 min mice were euthanized. Temperature drop from the baseline was calculated by subtracting each temperature with the baseline temperature without challenge. Mice injected with PBS vehicle showed no significant changes in body temperature (± 1°C).

#### Ovalbumin-induced itch

To test the behavioral response of itch, mice were sensitized intraperitoneally with 50 µg of ovalbumin (Ova) in 100 µL volume on Day 0, 20 µg on Day 11 and challenged with 50 µg of Ova or vehicle (PBS) on day 19 intradermally into the cheek in 10 µL volume, and bouts of scratching for a period of 30 min was quantified as described (19). For behavior experiments prior to the injection, mice were subjected to acclimatization period in the test chamber for 10 min on the day of the experiment. Ova or PBS was injected into the cheek, and scratching behavior was recorded for 30 min. Continuous scratching movement at the injection site utilizing hind paw was defined and recorded as a bout of scratching. The number of scratching bouts during the 30 min observation period was quantified by counting in slow motion frame by frame using Elmedia video player.

#### Statistical analysis

To maintain and ensure reproducibility and scientific rigor, 6-10 mice per experiment were used for all *in vivo* experiments. No sex dependent response has been reported yet and thus equal number of males and females were used. Results are represented as mean ± standard error of the mean (SEM) values. Different batches of cells culture (at least 3) were used in all *in vitro* experiments repeated 3-4 times ran in triplicates. For calcium mobilization, SEM has been omitted from figure for clarity. Statistical significance was determined by two-way ANOVA with multiple comparison and were set at *P ≤ 0.05, **P ≤ 0.01, ***P ≤ 0.001 and ****P ≤ 0.0001 analyzed by GraphPad Prism version 9. The statistical comparison that resulted in non-significant p-values were omitted from the graph for more clarity.

## Results

**Figure 1:**
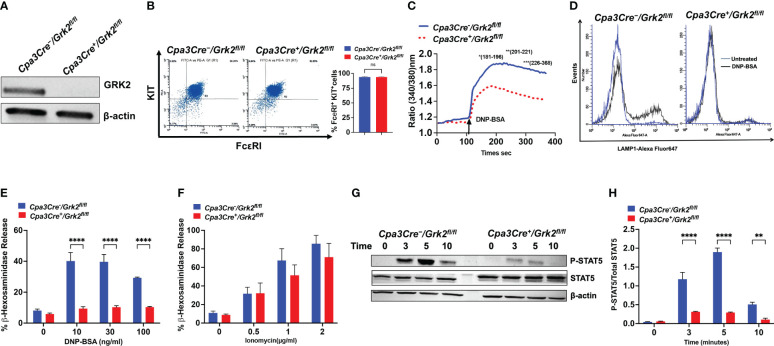
*Grk2*-deletion inhibits FcεRI-induced primary lung-derived MCs (PLMCs) activation. In PLMCs generated from *Cpa3Cre^-^/Grk2^fl/fl^* and *Cpa3Cre^+^/Grk2^fl/fl^* mice, **(A)** Western blotting for GRK2 protein expression with β-actin as loading control, **(B)** cell surface expression of FcεRI and KIT receptors as determined by flow cytometry; DNP-BSA-induced **(C)** calcium mobilization (10 ng/ml), **(D)** lysosomal-associated membrane protein1 (LAMP-1) expression (10 ng/ml) and **(E)** β-hexosaminidase release (0 -100 ng/ml) in cells primed with DNP-specific IgE (1 µg/mL, 16 h); **(F)** PMA (50 nM)-Ionomycin (0-2 µg/ml)-induced β-hexosaminidase release; **(G)** Immunoblots for P-STAT5 and STAT5 expression and **(H)** quantification of intensities (P-STAT5/STAT5) upon DNP-BSA (30 ng/ml; 0, 3, 5,10 min) challenge. Each experiment was performed 3-4 times at least in 3 different batches of cells. Error bars are standard error of mean (SEM) and significant differences were set at **p ≤ 0.01 and ****p ≤ 0.0001.

To investigate the role of GRK2 on FcεRI and MRGPRB2-mediated responses, we generated mice with MC-specific deletion of *Grk2* by crossing *Cpa3Cre* mice with *Grk2^fl/fl^* mice. Zhong et al. (17), showed that incubation of finely chopped mouse upper airway fragments with SCF and IL-3 results in the generation of primary lung-derived MCs (PLMCs), which express functional FcεRI. We therefore generated mouse PLMCs from *Cpa3Cre^-^/Grk2^fl/fl^* and *Cpa3Cre^+^/Grk2^fl/fl^* mice. Using Western blotting, we confirmed that GRK2 is absent in PLMCs generated from *Cpa3Cre^+^/Grk2^fl/fl^* mice (**Figure 1A**). Flow cytometry experiment with FcεRI and KIT antibodies demonstrated that absence of GRK2 has no effect on the differentiation of PLMCs (**Figure 1B**). However, the absence of GRK2 resulted in significant inhibition of IgE-mediated calcium mobilization (**Figure 1C**) and degranulation as assessed by cell surface expression of Lysosomal-associated membrane protein 1 (LAMP-1) (**Figure 1D**) and β-hexosaminidase release (**Figure 1E**). By contrast, degranulation induced by phorbol 12-myristate 13-acetate/Ca^2+^ ionophore was unaffected suggesting that the inhibitory effect of GRK2 on IgE-mediated degranulation results from the modification of a signaling component upstream of calcium mobilization (**Figure 1F**).

Signal Transducers and Activators of Transcription (STATs) are ubiquitously expressed cytoplasmic proteins and are well documented to play an important role in the development, survival, and proliferation of MCs (20, 21). It was recently demonstrated that tyrosine phosphorylation of STAT5 contributes to IgE-mediated signaling in MCs (22, 23). Given that absence of GRK2 and STAT5 (24) display similar IgE-mediated *in vitro* MC response, we hypothesized that GRK2 could contribute to FcεRI signaling by promoting tyrosine phosphorylation of STAT5. As per previous report (22), we found that activation of control cells with antigen/IgE resulted in robust but transient tyrosine phosphorylation of STAT5. However, the absence of GRK2 resulted in significant inhibition of STAT5 phosphorylation (**Figures 1G, H**).

**Figure 2:**
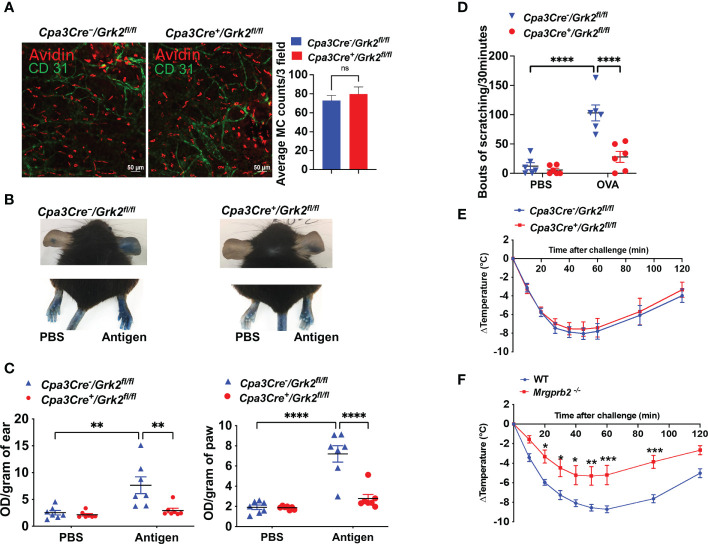
*Grk2*-deletion inhibits IgE-mediated anaphylaxis and itch but not passive systemic anaphylaxis (PSA). In *Cpa3Cre^-^/Grk2^fl/fl^* and *Cpa3Cre^+^/Grk2^fl/fl^* mice, **(A)** Confocal images of ear stained with Texas Red-conjugate Avidin (Mast Cells) and Alexa Fluor488-conjugated anti-CD31 antibody (Endothelial cells); Measurement of vascular permeability in passively sensitized mice (n=6-7) with DNP-specific IgE (20 ng, right ear/hind paw) or PBS (left ear/hind paw) intravenously challenged with DNP-BSA (20 µg in Evans blue). **(B, C)** Representative images and quantification of Evans blue extravasation; **(D)** Bouts of scratching (30 min) in ovalbumin-sensitized (Day 0: 50 µg; Day 11: 20 µg) and challenged (Day 19: 50 µg, cheek) mice. Measurement of core body temperature drop in passively sensitized **(E)** *Cpa3Cre^-^/Grk2^fl/fl^* and *Cpa3Cre^+^/Grk2^fl/fl^* (n=8) and **(F)** Wild-type and *Mrgprb2^-/-^* mice (n=7-9) with DNP-specific IgE (20 µg, intraperitoneal, 24 hours) and challenged with DNP-BSA (200 µg). Error bars are standard error of mean (SEM) and significant differences were set at *p ≤ 0.05, **p ≤ 0.01, ***p ≤ 0.001 and ****p ≤ 0.0001.

Immunofluorescent staining of dermal MCs in mouse ear with Texas Red Avidin (MCs) and Alexa Fluor 488-conjugated CD31 (endothelial cells) revealed that GRK2 has no effect on the number and distribution of MCs adjoining blood vessels (**Figure 2A**). We next sought to determine the effects of *Grk2*-deletion on IgE-mediated passive cutaneous anaphylaxis (PCA) and itch *in vivo*. For PCA, *Cpa3Cre^-^/Grk2^fl/fl^* and *Cpa3Cre^+^/Grk2^fl/fl^* mice were sensitized with intradermal injection of DNP-specific IgE (right ear/paw) or PBS vehicle (left ear/paw), and after 24 h, mice were challenged with an intravenous injection of DNP-BSA in 1% Evans blue, and local vascular permeability was measured. As shown in **Figures 2B, C**, *Cpa3Cre^+^/Grk2^fl/fl^* mice displayed significantly reduced vascular permeability compared to control mice in both experimental tissue locations (ear and paw). For itch studies, mice were sensitized intraperitoneally with ovalbumin (Ova) on Day 0 and 11 and challenged with Ova on day 19 intradermally into the cheek, and bouts of scratching for a period of 30 minutes was quantified (19). As shown in **Figure 2D**, *Cpa3Cre^+^/Grk2^fl/fl^* mice displayed significantly reduced number of scratching compared to control mice. We further tested the effect of *Grk2*-deletion on IgE-mediated systemic anaphylactic response utilizing the PSA model. For this, *Cpa3Cre^-^/Grk2^fl/fl^* and *Cpa3Cre^+^/Grk2^fl/fl^* mice were sensitized with intraperitoneal injection of DNP-specific IgE and after 24 h challenged with an intraperitoneal injection of DNP-BSA. Core body temperature was measured with a rectal thermometer every 10 min for 60 min and every 30 min after that until 120 min and temperature drop from the baseline was assessed. To our surprise, we found that unlike PCA and itch, *Grk2*-deletion had no effect on PSA (**Figure 2E**).

Until recently, IgE-mediated MC activation were thought to contribute to atopic dermatitis and allergic contact dermatitis, but it is now realized that MRGPRB2 plays an important role in these responses through its activation by agonists generated from nerve endings (substance P) and keratinocytes (proadrenomedullin N-terminal peptides), respectively (8, 19). Although MRGPRB2 was originally thought to be expressed mostly in skin MCs, transcripts for this receptor are also found in fat, nasopharyngeal, peritoneal and colon MCs (25, 26). We, therefore, hypothesized that MRGPRB2 could contribute to IgE-mediated systemic anaphylaxis. Indeed, we found that IgE-mediated PSA was significantly reduced in *Mrgprb2^-/-^* mice compared to control mice (**Figure 2F**). This raises the interesting possibility that IgE-mediated MC activation leads to the secretion of endogenous agonists that activate MRGPRB2, thus suggesting a plausible mechanism for the role of this receptor in PSA. However, the ligand involved is not known.

**Figure 3:**
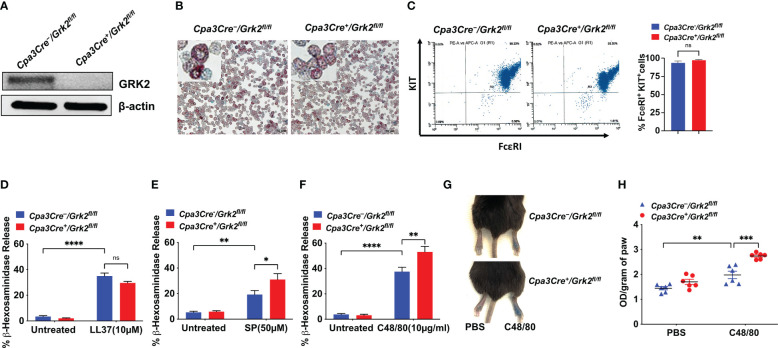
*Grk2*-deletion has no effect on LL-37-induced degranulation but enhances the response to Substance P (SP) and Compound 48/80 (C48/80). In peritoneal MC (PMCs) from *Cpa3Cre^-^/Grk2^fl/fl^* and *Cpa3Cre^+^/Grk2^fl/fl^* mice, **(A)** Western blotting for GRK2 protein expression with β-actin as loading control, **(B)** alcian/safranin staining, **(C)** cell surface expression of FcεRI and KIT receptors as determined by flow cytometry; β-hexosaminidase release induced by **(D)** LL-37 (10 µM), **(E)** SP (50 µM) **(F)** C48/80 (10 µg/ml); Each experiment was performed 3-4 times at least in 3 different batches of cells. **(G, H)** Evans blue extravasation after intradermal-injection with C48/80 (100 ng) in *Cpa3Cre^-^/Grk2^fl/fl^* and *Cpa3Cre^+^/Grk2^fl/fl^* mice (n=6). Error bars are standard error of mean (SEM) and significant differences were set at *p ≤ 0.05, **p ≤ 0.01, ***p ≤ 0.001 and ****p ≤ 0.0001.

MRGPRX2 agonists are categorized into G-protein biased or balanced based on downstream activation (13–15). The role of GRK2 in such regulation is not known. Thus, we selected G protein-biased (LL-37) and balanced agonists (C48/80 and SP) to assess if GRK2 differentially regulates degranulation response induced by these agonists. Unlike PLMCs, cells cultured from the mouse peritoneum (PMCs) expresses functional MRGPRB2 (27). We, therefore, performed *in vitro* studies with PMCs cultured from peritoneal lavage cells of *Grk2*-deleted mice (*Cpa3Cre^+^/Grk2^fl/fl^*) and their control littermates (*Cpa3Cre^-^/Grk2^fl/fl^*). We found that the absence of GRK2 (**Figure 3A**) had no effect in alcian/safranin staining property (**Figure 3B**) or cell surface expression of FcεRI and KIT (**Figure 3C**). Furthermore, absence of GRK2 had no effect on LL-37-induced degranulation in mouse PMCs (**Figure 3D**), which is consistent with our previous report that silencing the expression of GRK2 in a human MC line endogenously expressing MRGPRX2 has no effect on LL-37-induced degranulation (11).

In contrast to LL-37, SP- and C48/80-induced degranulation was significantly enhanced in the absence of GRK2 (**Figures 3E, F**). This enhanced response to SP and C48/80 is unlikely due to increased MRGPRB2 expression as degranulation in response to LL-37 remained unaffected in the absence of GRK2. Based on our previous findings that SP and C48/80 cause MRGPRX2 desensitization and receptor internalization (12, 14, 15), it is likely that GRK2 phosphorylates Ser/Thr on MRGPRB2 and the enhanced response in the absence of GRK2 reflects a lack of receptor phosphorylation and desensitization. To determine the *in vivo* consequence of *Grk2* deletion, we intradermally injected C48/80 (right) or PBS vehicle (left) in the hind paw of *Cpa3Cre^-^/Grk2^fl/fl^* and *Cpa3Cre^+^/Grk2^fl/fl^* mice following the intravenous injection of Evans blue dye. Consistent with C48/80-induced degranulation in mouse PMCs, there was a significant enhancement of vascular permeability, as measured by dye extravasation, in *Cpa3Cre^+^/Grk2^fl/fl^* mice when compared to control mice (**Figures 3G, H**). These findings clearly demonstrate that the ability of GRK2 to modulate MRGPRB2-mediated MC degranulation depends on the agonist used for activation.

## Discussion

GRK2 is a Ser/Thr kinase that phosphorylates agonist-stimulated GPCRs to promote their desensitization and internalization (3, 10). However, we recently showed that GRK2 contributes to FcεRI (a non-GPCR)-mediated MC responses (5, 28), but the mechanism and *in vivo* relevance is unknown. We have previously shown that C48/80 and SP serve as balanced agonists for MRGPRX2 and promote desensitization and receptor internalization (14, 15). By contrast, LL-37 serves as a G protein-biased agonist for MRGPRX2 and that silencing GRK2 expression in a human MC line (LAD2 cells) has no effect on degranulation in response to this agonist (11, 29). However, the consequence of *Grk2*-deletion on MRGPRB2-mediated degranulation in response to C48/80, SP and LL-37 is not known. In this study, we demonstrated that while GRK2 promotes IgE-mediated calcium mobilization and degranulation in PLMCs *in vitro via* the tyrosine phosphorylation of STAT5, it promotes local cutaneous anaphylaxis but not systemic anaphylaxis. Interestingly, we found a previously unknown role for MRGPRB2 in IgE-mediated systemic anaphylaxis. We also demonstrated that GRK2 does not regulate MRGPRB2-mediated degranulation in response to LL-37.

Pullen et al. (22), reported a novel observation that Fyn-dependent tyrosine phosphorylation of STAT5 is required for IgE-mediated MC function and that these responses are independent of canonical Syk and ERK activation. Additionally, STAT5-deficient mice demonstrated reduced MC degranulation *in vivo*, a similar phenotype that we observed in *Grk2*-deleted mice. STAT5 regulation in IgE-mediated signaling is poorly understood. Thus, this novel role of GRK2 in IgE-mediated STAT5 activation is intriguing. GRK2 is a Ser/Thr kinase, thus direct tyrosine phosphorylation of STAT5 by GRK2 is unlikely. STAT5 co-localizes with FcεRI, and its activity is controlled by Fyn kinase and the negative regulators, Gab2 and SH2 domain-containing phosphatase-1 (SHP-1) (22). Thus, it is possible that GRK2 either acts as an adaptor protein that directly associates with the receptor to facilitate the recruitment of STAT5 to promote Fyn activation or it serves to block the inhibitory activity of Gab2 and SHP-1. These possibilities will be subject of our future investigations.

Although aggregation of FcεRI on MCs provides a well-established mechanism for atopic and hypersensitivity reactions, recent evidence suggests that MRGPRB2 activation by SP contributes to atopic dermatitis (8), which was previously thought to be modulated *via* IgE-mediated pathway (1). In the present study, we have shown that absence of GRK2 enhances SP-induced MRGPRB2-mediated MC degranulation. This raises the interesting possibility that unlike IgE-mediated PCA and itch, GRK2 provides an inhibitory signal for atopic dermatitis through MRGPRX2/B2 desensitization.

Although experimental PCA and PSA in mice are initiated through IgE sensitization and activation of MCs, there appears to be differences in requirement for GPCRs. Thus, while MRGPRB2 does not participate in PCA (7), sphingosine-1-phosphate released from activated MCs activates its cognate GPCR and contributes to PSA (9). The present study provides the first demonstration that MRGPRB2 contributes to IgE-mediated PSA and that GRK2 does not regulate this response. These findings suggests that IgE-mediated MC activation in PSA results in the release of G protein-biased MRGPRB2 agonists, which in turn activate MCs resulting in hypothermia. LL-37 is a G protein-biased agonist for MRGPRX2 (11, 29), which is released from activated MCs and epithelial cells (30–32). We recently showed that LL-37-induced degranulation in mouse PMCs is substantially reduced in the absence of MRGPRB2 (33) and the present study demonstrated that absence of GRK2 has no effect on LL-37-induced degranulation. The possibility that LL-37 or other endogenously derived MRGPRB2 agonist that contribute to IgE-mediated PSA remains to be determined.

In summary, we have shown that GRK2 contributes to IgE-mediated PCA and itch likely *via* the formation of a signaling complex with FcεRI, Fyn, STAT5, Gab2 and SHP-1. Thus, disrupting the interaction of GRK2 with these signaling proteins may provide a new approach to modulate these responses. An intriguing finding of this study is that MRGPRB2 is required for IgE-mediated systemic anaphylaxis and that this response is not modulated by GRK2. Humanized mice have been used to study the role of human MCs in PCA and PSA (34). Furthermore, specific MRGPRX2 antagonists that do not inhibit FcεRI or MRGPRB2-mediated MC degranulation have been developed (35). These reagents could be utilized in the future to determine the role of MRGPRX2 in systemic anaphylaxis, including food allergy.

## Data availability statement

The raw data supporting the conclusions of this article will be made available by the authors, without undue reservation.

## Ethics statement

The animal study was reviewed and approved by Institutional Animal Care and Use Committee at The University of Pennsylvania.

## Author contributions

HA contributed to conception, supervision, and funding acquisition of the study. MT contributed to the conception, performed the experiments and analyzed the data and wrote the first draft of the manuscript. All authors contributed to manuscript revision, read, and approved the final manuscript. All authors contributed to the article and approved the submitted version.
